# A Comprehensive Review of the Immunological Response against Foot-and-Mouth Disease Virus Infection and Its Evasion Mechanisms

**DOI:** 10.3390/vaccines8040764

**Published:** 2020-12-14

**Authors:** Ibett Rodríguez-Habibe, Carmen Celis-Giraldo, Manuel Elkin Patarroyo, Catalina Avendaño, Manuel Alfonso Patarroyo

**Affiliations:** 1 Animal Science Faculty, Universidad de Ciencias Aplicadas y Ambientales (U.D.C.A), Bogotá 111166, Colombia; ibetrodriguez@udca.edu.co (I.R.-H.); ccelis@udca.edu.co (C.C.-G.); 2 Masters Programme in Veterinary Science, Universidad de Ciencias Aplicadas y Ambientales (U.D.C.A), Bogotá 111166, Colombia; 3 Fundación Instituto de Inmunología de Colombia (FIDIC), Bogotá 111321, Colombia; mepatarr@gmail.com; 4 Faculty of Medicine, Universidad Nacional de Colombia, Bogotá 111321, Colombia; 5 School of Medicine and Health Sciences, Universidad del Rosario, Bogotá 112111, Colombia

**Keywords:** foot-and-mouth-disease virus, immune response, immune evasion mechanism

## Abstract

Foot-and-mouth disease (FMD) is a highly contagious viral disease, which has been reported for over 100 years, and against which the struggle has lasted for the same amount of time. It affects individuals from the order Artiodactyla, such as cattle, swine, sheep, wild animals from this order, and a few non-cloven hoofed species, such as mice and elephants. FMD causes large-scale economic losses for agricultural production systems; morbidity is almost 100% in an affected population, accompanied by a high mortality rate in young animals due to myocarditis or an inability to suckle if a mother is ill. The aetiological agent is an *Aphthovirus* from the family Picornaviridae, having seven serotypes: A, O, C, SAT1, SAT2, SAT3, and Asia 1. Serotype variability means that an immune response is serospecific and vaccines are thus designed to protect against each serotype independently. A host’s adaptive immune response is key in defence against pathogens; however, this virus uses successful strategies (along with most microorganisms) enabling it to evade a host’s immune system to rapidly and efficiently establish itself within such host, and thus remain there. This review has been aimed at an in-depth analysis of the immune response in cattle and swine regarding FMD virus, the possible evasion mechanisms used by the virus and describing some immunological differences regarding these species. Such aspects can provide pertinent knowledge for developing new FMD control and prevention strategies.

## 1. Introduction

The immunological system protects organisms against colonization by pathogenic agents and promotes tissue repair following injury. It uses various mechanisms, such as successive defence layers or barriers, each being more specific and specialized than the previous one. Such mechanisms can be grouped into innate immunity and acquired immunity. Concerning innate immunity, the first barrier consists of physical or anatomical barriers like the skin and the mucous membranes covering the respiratory, digestive and reproductive tracts; the second barrier contains phagocytic and non-phagocytic cells which respond rapidly but non-specifically when pathogens have managed to surmount the first barriers. B- and T-lymphocyte action forms part of the adaptive immune system providing a more specific though slower response. It is worth stressing that innate and adaptive immune systems depend on each other [1,2,3,4].

Many pathogens’ survival depends on them establishing themselves in a particular host, either in body cavities (i.e., gastrointestinal parasites, such as helminths) or within cells (i.e., some protozoa, viruses, and bacteria). Pathogens and their hosts have evolved jointly; whilst pathogens can accumulate several mutations per generation (that usually involves few hours), the hosts can counteract the attack through their ability to generate a huge number of T- and B-cell clones with different antigen specificities [2,5]. In spite of this, viral pathogens particularly mutate rapidly and evolve to counteract mammals’ non-specific innate responses and their highly specific adaptive immune response [6].

Foot-and-mouth disease (FMD) is a highly infectious viral disease affecting animals from the order Artiodactyla (even-toed hoofed animals/ungulates) such as cattle, swine, sheep, and goats and wild hoofed animals, such as deer, antelopes, buffalo, bison, reindeer, and giraffes. Old World camelids, such as camels and dromedaries, and ones from the New World like llamas, alpacas and vicuña have been experimentally infected but there are no reports of them becoming infected in their native environments. Species which do not belong to this order have been shown to be susceptible to the virus, i.e., hedgehogs, armadillos, chiguiros, kangaroos, rats, and mice; captive Asian and African elephants have been infected but there have been no reports of them becoming diseased in the wild [7,8,9]. FMD is endemic in South America, Africa, Asia, and parts of Europe, causing largescale economic losses; ulcers form in the animals’ mouths and on their hooves and teats and involves loss of body condition. This leads to exorbitant spending on veterinary drugs, along with foot-and-mouth disease-free countries rejecting the import of livestock and livestock-derived products from endemic countries and/or those affected by FMD [10,11]. FMD is on the World Organization for Animal Health’s Terrestrial Animal Health Code list and it must be declared. It is the first disease regarding which this organization has issued an official list of countries or zones certified as being free of the disease with or without vaccination.

FMD is produced by a virus belonging to the family Picornaviridae, genus *Aphthovirus*; it is a positive-sense, single-strand RNA virus. It has ~8300 nucleotides, included in a icosahedral protein capsid, formed by protomers integrated by four structurally different proteins: VP1, VP2, VP3, and VP4 [12,13]. Seven immunologically different types can be differentiated by serological tests; they are known as A, O, C, South African-Type 1 (SAT-1), South African-Type 2 (SAT-2), South African-Type 3 (SAT-3), and Asia 1 [10,11].

Serospecific and cyclic mass vaccination is used by most nations when trying to control and eradicate the virus [10,14,15]. However, the virus’ enormous genetic variability and antigenic diversity has led to its population structure being defined as a quasi-species [16,17], thereby hampering its control by vaccination [16,18].

A better understanding of virus–host interaction is essential for developing strategies (i.e., antiviral approaches and novel vaccines) able to rapidly control the disease and reduce the negative impact on the economy, bearing in mind the evasion mechanisms used by the virus for escaping the immune response [19], this being the purpose of this review.

## 2. Foot-and-Mouth Disease Virus Pathogenesis

The virus’ pathogenic mechanism can be analyzed as a chain of interactions between virus and host. The specific steps involved may vary depending on the type of virus and host; however, to explain infection developing with the virus, a general model can be described which is applicable to most cases:

1. Entry to a susceptible host;
2. Replication for increasing viral load;
3. Dissemination from the entry site to the tissues and/or target organs, developing the infection, and damage to cells and organs;
4. Elimination, contamination, and dissemination to the environment;
5. Persistence in the environment; and
6. Transmission to new hosts and beginning a new cycle [5].

### 2.1. Foot-and-Mouth Disease Virus Entry to a Host

Foot-and-mouth disease virus (FMDV) usually enters an animal by aerial route, the respiratory tract being the main infection route. The virus enters as aerosols produced by animals coughing or sneezing, infected swine being the most important source for producing them, followed by cattle and sheep; high infectivity indices have been found in aerosols from milk and feces [20].

FMDV can also enter via the oral cavity by mechanically transmitting infectious nasal secretions (nose–nose contact) or in saliva (by licking or the shared use of contaminated utensils: watering and feeding devices, etc.). Fortuitous entry is enabled by injury to the skin in skin folds (such as, interdigital areas) since intact skin has active mechanisms related to its acid pH, the presence of antibacterial fatty acids and normal flora, making the skin able to resist many pathogens’ aggression [4,21,22].

Few viral particles are usually needed for infection to begin in most species, except in swine since this specie is more resistant to infection by respiratory route than cattle or sheep; nevertheless, it is much more sensitive to infection by oral route. Cattle can thus become infected by 5 to 10 viral particles, sheep by 15 to 20 particles whereas a much greater amount is required to infect swine by respiratory route [12]. Experimental studies, like that by Alexandersen et al. [22] required ten 50% tissue culture infective doses (TCID_50_) for infecting ruminants compared to 10^3^ TCID_50_ required for infecting swine. Likewise, viral particle dissemination volume was greater in swine which can disseminate 10^8^ viral particles, i.e., equivalent to 3000 times more particles than that for cattle or sheep during the same period of time [12,23,24,25].

### 2.2. Foot-and-Mouth Disease Virus Entry to Cells

Once FMDV has entered a host it needs to establish and/or disseminate itself; viruses must bind to and infect cells on some body surfaces since they are obligate intracellular microorganisms [26]. FMDV has tropism for epithelial cells in which it replicates rapidly from the same entry point, giving rise to vesicles called primary canker sores which usually pass unnoticed [27]. FMDV particles enter cells through receptor-mediated endocytosis; this begins with the interaction between the virus’s proteins binding to cell surface receptors [28]. Viral particles can use different receptors during viral pathogenesis’ different stages. FMDV is the only virus from the family Picornaviridae which can use four integrins (αvβ1, αvβ3, αvβ6, and αvβ8) for mediating infection; however, each receptor’s function during cell infection by FMDV has still not been elucidated [29]. It has been documented that integrins a5β and avβ5 are involved in cell susceptibility to FMDV infection [28], thereby increasing the amount of integrins used by the virus to infect epithelial cells (from 4 to 6). Molecular and immunodiagnostic studies have confirmed avβ6’s role as cell receptor by showing that this integrin’s expression is restricted to epithelial cells (FMDV’s infection target) located in the upper airways’ epithelium, oral cavity, gastrointestinal tract, and/or the hooves’ coronary bands. Relevantly, it is expressed in in high levels in the airways tonsillar crypt epithelium in sheep and cattle, the primary site for viral replication [30,31].

It has also been observed in vitro that FMDV is capable of using heparan sulphate (HS) as receptor [29]. It was initially considered that HS was a serotype O co-receptor but later studies have shown that serotypes A, C, Asia 1, and SAT 1 can also bind to HS, thereby facilitating their entry to cells, even though their peptides and/or residues interacting with HS are located in different parts of VP1 or VP3 capsid proteins [32,33,34,35].

**Figure 1:**
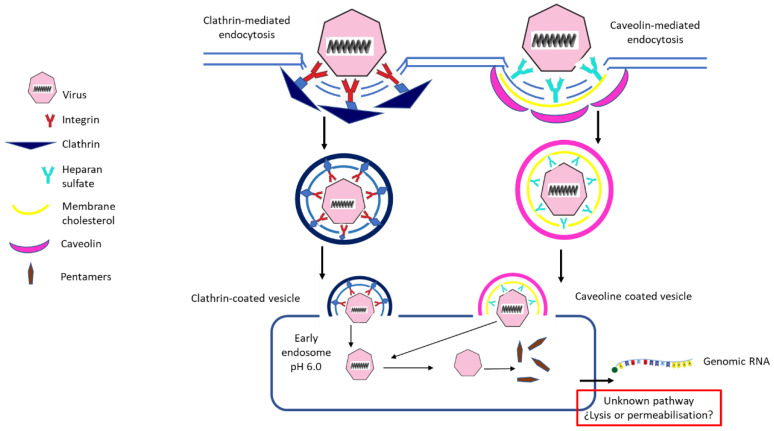
A scheme showing Foot-and-mouth disease virus (FMDV) entry to target cells. Adapted [36].

Following integrin binding to cell membrane, FMDV infects target cells by clathrin-mediated endocytosis and then becomes mobilized via early endosomes. If binding occurs via HS, the virus enters cells by caveolin-mediated endocytosis, followed by early endosome use (Figure 1). It has been demonstrated that clathrin-mediated endocytosis develops faster than caveolin-mediated endocytosis [33,36].

Some studies have suggested that cattle and swine dendritic cells (DC) are susceptible to FMDV infection; however, such experiments were made when little was known about ruminant DC and data should thus be interpreted with caution [37]. It has been described that FMDV adsorption by macrophages and DC could be mediated by the crystallisable fraction (Fc) receptor (FcR), an additional mechanism which could maintain pathogen activity and may be used for infection persistence in some animals (an interesting aspect still being studied) [37,38].

### 2.3. Foot-and-Mouth Disease Virus Replication, Dissemination, and Effect on Target Organs

Once the virus is inside an endosome, this compartment’s low pH (6.0 to 5.5 pH late endosome, 6.5 to 6.0 early endosome) triggers the chemical stripping and release of the viral genome, which translocates to the cytosol through the endosome membrane [39]; such translocation is *cap*-independent [40]. Genomic RNA’s (gRNA) positive polarity functions as messenger RNA (mRNA), is intrinsically infectious. The 5′ extreme has a protein called genome-associated viral protein (gVP) followed by the 5′ UTR region (around 834 nucleotides-long) having a cytidine-rich region (poly(C)) and the internal ribosome entry site (IRES) binding directly to the ribosomes, followed by the open reading frame (ORF). The 3′ UTR region is located between the stop codon and a variable length poly(A) tail. One RNA molecule is sufficient for initiating infection, implying that it could function as translation template so that polymerase can be produced along with RNA replication [41,42,43].

RNA can begin its translation in 2 AUG codons located in the L proteinase (L^pro^) amino terminus, enabling the encoding of fifteen proteins: four structural ones forming the viral capsid and eleven non-structural ones needed for viral replication and inhibiting some host cell functions [19,40,44].

**Figure 2:**
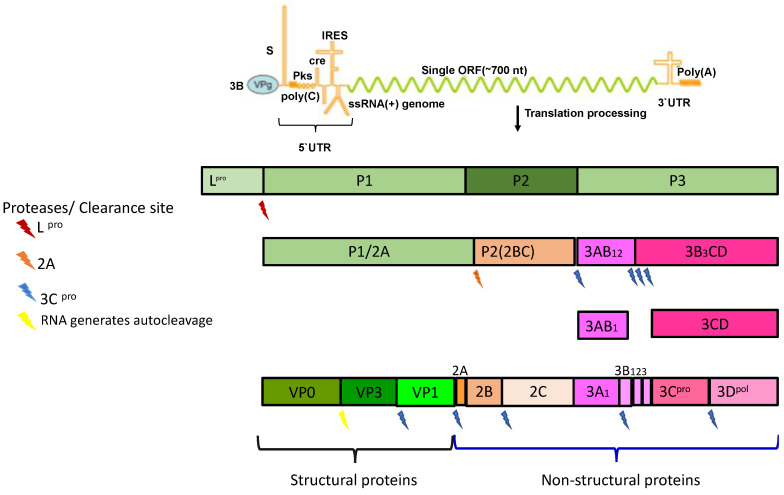
Diagram of the FMDV genome, viral polypeptide processing, and structural and non-structural protein formation. Adapted [14,36,42,43].

Proteolytic cleavage occurs during viral polyprotein processing, during which determined protein precursors are released: L proteinase (L^pro^) and three polypeptides—P1, P2 and P3 [45]. P1 encodes structural proteins VP1, VP2, VP3, and VP4 which are assembled to form the viral capsid whilst P2 encodes three non-structural proteins 2A, 2B, and 2C [45,46]. P3 encodes four non-structural proteins: 3A, 3B, 3C, and 3D. P3A is membrane-associated in the replication complex; its function regarding viral genome replication has yet to be elucidated. P3B binds to the viral genome and is called VPg. P3C^pro^ is a viral protease responsible for post-translational modifications of viral proteins and P3D^pol^ functions as RNA-dependent RNA polymerase responsible for viral RNA replication [47] (Figure 2).

FMDV replication is a rapid and efficient process; chromatin condensation can be observed in host cells one hour post-infection (pi), membrane proliferation within infected cells three hours pi and host cell lysis and new viral output six hours pi [30,48].

The first viral replication occurs following natural infection, mainly in oropharyngeal cells, leading to some vesicles called primary canker sores which usually pass unnoticed [12,23]. Following the first replication, the virus passes into the bloodstream; the viremia phase becomes developed, characterized by an animal’s high temperature and general malaise. FMDV undergoes a second replication in the reticuloendothelial cells and the parenchyma of target organs (liver, spleen, bone marrow, and striated muscle) during this period. The virus returns to the epithelial cells in the snout, hooves, and mammary glands, producing the secondary vesicles characteristic of the disease. The mechanism by which viral particles pass from the blood to not very vascularized areas of the epithelium has not yet been elucidated; it may be associated with migration to infected macrophage tissue or the amount of infectious particles entering a host [35,38,49,50,51].

### 2.4. Immune Response

#### 2.4.1. Innate Response

Inhaled particles which might affect the respiratory tract are size-filtered; those greater than 10 μm remain in the mucocilliary layer in the nasal cavity and upper airways, whilst those smaller than 5 μm can go directly to the distal airspaces where they become phagocyted by the alveolar macrophages. Most inhaled virions are retained by the mucus and transported by the action of the cilia from the nasal cavity and upper airways to the pharynx to be swallowed or eliminated by coughing [3].

The respiratory tract’s mucosal surfaces are covered by epithelial cells which can sustain viral infection; defence mechanisms associated with local defence and humoral and cellular immunity are thus needed to minimize the risk of infection [4]. The mucocilliary membrane is one such local defence mechanism; it extends from the nasal cavity to the distal airway in the lungs. It consists of a layer of mucus which cleans the nostrils and supresses irritants and harmful agents. The respiratory system has two macrophage types; the former, located in the interstitium, and the alveolar ones located on alveoli surface to protect them. Both fulfil defence functions against pathogens, such as the phagocytic uptake of inhaled particles and (less efficiently) bacterial phagocytosis. They are allergen destroyers and act as T-cell antigen presenters, releasing mediators enabling inflammation to begin and controlling and remodelling and repairing lung tissue [52,53,54,55].

PRRs interacting with their corresponding PAMPs activate phagocytosis or signalling pathways which stimulate cytokine production, the expression of adhesion molecules and co-stimulators. Toll-like receptors (TLR) have been the respiratory system PRRs studied in the greatest depth to date. They have been classified as transmembrane, type 1, membrane receptors; they have an ectodomain consisting of leucine-rich repeats (LRR), which are responsible for recognising PAMPs, and cytoplasmatic domain, homologous to the IL-1 receptor cytoplasmic region known as TIR domain necessary for downstream signalling [56,57]. Greater TLR expression has been identified in the macrophages, DC, B-lymphocytes, and respiratory tract mucosa epithelial cells.

Evaluating respiratory system response has shown that respiratory tract mucosa epithelial cells are highly susceptible to direct infection by the virus due to the integrins, specifically αvβ6. Zhu et al. [58] established the relationship between these integrins and IL-1 secretion by macrophages, monocytes and DC as a response to tumor necrosis factor (TNF). IL-1 forms part of the initial immune response (fever, T-lymphocyte, and macrophage activation) intervening in rapid extracellular matrix renewal and playing a key role in vesicular lesion pathogenesis, leading to the formation of canker sores [55,58,59,60].

TLR induction activates the signalling control pathways, in turn activating antimicrobial genes, inflammatory cytokine production inducing leucocyte migration (chemotaxis), specifically neutrophils, provoking more DC to mature, thereby inducing more co-stimulatory molecules and increasing their antigen-presenting capability. TLRs thus participate in developing the direct adaptive immunity response against pathogens [4,55,59].

The members of this family (TLR) have been classified into two subgroups according to their location; ten TLRs populations have been described in cattle; TLR1, TLR2, TLR4, TLR5, TLR6, and TLR11 are exclusively expressed on cell surface and TLR3, TLR7, TLR8, and TLR9 are located in intracellular vesicles, such as endosomes, lysosomes or the endoplasmic reticulum. Such intracellular TLRs act as viral nucleic acid detectors and activate the innate immune system’s anti-viral response [61]. TLR2 expression has been seen in cells infected by native virus and cells transfected by a DNA vaccine against FMDV [62,63]. DC and macrophages express TLR3 and express it more forcefully in CD8α^+^ DCs having high affinity for virus-infected apoptotic bodies. Such expression may also occur in FMDV infection [61]

The mucocilliary barrier was mentioned as a local defence mechanism at the start of this section; the mucus secreted by the glands forms part of this. It is made up of 95% water, 4% mucins (high molecular weight glycoproteins conferring viscosity and elasticity), a series of protection factors (specific ones forming humoral immunity), and immunoglobulins (mainly mucosal secretory IgA (SIgA) (80% of immunoglobulins in nasal mucus are SIgA) and systemic circulation IgG, both contributing to antiviral defence in the lower respiratory airways and in the mucus). Non-specific protection factors such as lysozymes, lactoferrin, and interferon (IFN) are found to a lesser extent [52].

This defence system also contains accumulations of non-encapsulated lymphatic tissue acting as secondary or peripheral immune response cell producers, i.e., mucosa-associated lymphoid tissue (MALT) being lymphocytic aggregates in the gastrointestinal mucosa, genital-urinary tract, and lower and upper respiratory tracts, the latter being called nasal-associated lymphoid tissue (NALT) [2,52]. The respiratory apparatus’ immune system also includes bronchus associated-lymphoid tissue (BALT), the tonsils and adenoids, and alveolar and bronchial macrophage-mediated cellular immunity. These aggregates function as B-lymphocyte proliferation and differentiation points following antigen stimulation [55].

DC and plasmacytoid DC (pDC) are the main antigen-presenting cells in mammals; they also regulate the function of B-, T-lymphocytes, and other immunity cells via cytokine secretion. DC produce significant amounts of IFN-α following viral infection; immunological studies in humans and mice have ascertained their function in the association between innate and adaptive immune responses, concluding that these cells can manipulate the immune system [37,64,65].

Considerable DC heterogeneity has been described in cleft hoof animals; characterising DC subsets, their role in the immune response and interaction with FMDV could help to clarify their importance as markers during infection [37,64,66]. In vitro studies by Guzylack-Piriou et al. [67] demonstrated that swine pDC were not susceptible to FMDV and did not produce IFN-α after being exposed to it, thereby providing the virus with an opportunity to replicate and spread. However, they also demonstrated that FMDV infects pDC and initiates an abortive replication cycle resulting in high IFN-α expression levels by forming immune complexes binding to FcγRII (IgG Fc receptor). They used these findings to suggest that when the levels of Abs specific against FMDV are low and incapable of inducing protection during the first stages of adaptive response they could still be sufficient for mediating pDC viral infection and induce the subsequent release of IFN-α needed for eliminating the virus [67].

Bautista et al. [68] evaluated the effect of FMDV on swine skin-derived DCs in vitro. Even though the virus infected the cells, no viral replication, viral protein production or viral progeny were seen in them, nor was there any phagocytic effect or cell surface co-stimulatory molecule expression; however, IFN-α and IFN-β were released. A reduction in IFN-α production ex vivo was observed three days pi in DC and monocytes isolated from swine when stimulated with viral and/or synthetic proteins. However, the rapid advance of FMDV infection in swine suggested that IFN levels were not high enough to trigger an antiviral response in neighboring epithelial cells and/or the viral titres produced by infected cells were not sufficient or efficient for stimulating INFα/β secretion by DCs [68].

Guzylac-Piriou et al. [67] showed that IFN-α secretion in swine did not just depend on FMDV activity in pigs’ pDC but also triggered a viral abortive replication cycle related to high IFN-α production when the virus was opsonized by IgG, infecting pDC via the FcγRII receptor (CD32). This could explain why animals having very low specific Ab levels could be protected in the short-term after vaccination. These pDCs’ function would then be to retard viral infection until Abs levels could become more effective and could promote protective immune responses [66,67].

Sei et al. [69] demonstrated that circulating DC and pDC frequency in cattle blood tissue increased, reaching a maximum peak 3 to 4 days pi following FMDV entry. They also observed that (unlike that reported by other authors) these cattle had lymphopenia, MHC class II expression in DC and pDC molecules became drastically reduced and there was increased IL-10 production by DC and monocytes; these values returned to normal following the elimination of FMDV from the blood stream. Such observations highlight that FMDV is able to suppress the beginning of an effective adaptive immune response, stimulating IL-10 production by DC and monocytes, reducing MHC II expression and inhibiting antigen processing by DCs, even though it stimulates an increase in DC and monocytes [69].

Interferons, like IFN-α, IFN-β, and IFN-γ, and other cytokines, like interleukin 6 (IL-6), IL-8, and IL-12, have been seen to be involved in recovering from the infection. Their plasma concentration becomes increased following vaccination or field challenge, inducing monocyte and/or macrophage activation, neutrophil chemotaxis, amplified local inflammation, and adhesion molecule regulation [70,71,72]. The immune response against viral infection begins when interferons are expressed; these induce immune cell migration to the infection site, so much so that interferons have been used as treatment for controlling such infection [49,73].

IFN-α inhibits FMDV replication and this cytokine’s release protects swine from subsequent infection; however, susceptible animals becoming infected by the virus develop viremia one to three days after infection and rapid clinical presentation of the disease, suggesting that the virus can block or overcome the initial IFN-α response produced by pDC and maybe other cells [74].

**Figure 3:**
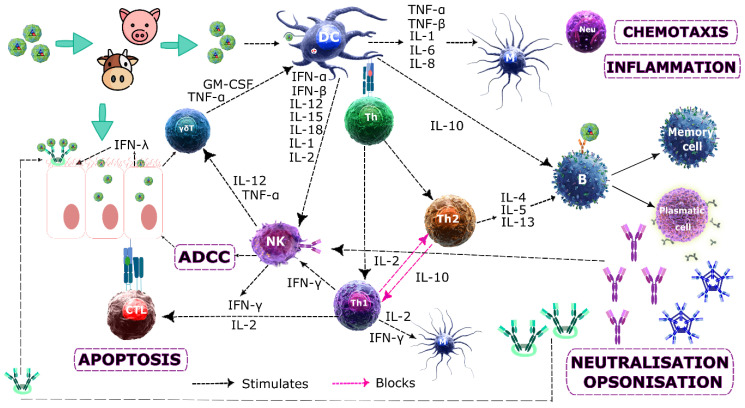
Ideal host immune response to FMDV. Anatomical, physiological, endocytic, phagocytic, and inflammatory barriers initially react against FMDV: the mucosity produced by respiratory tract ciliated cells retain part of the viral particles; neutrophils (Neu), eosinophils, dendritic cells (DC), macrophages (M), and cytotoxic (CTL) and natural killer (NK) lymphocytes, complement proteins and other inflammation mediators begin to act. Stress signals trigger gdT in tissue to secrete cytokines, thereby attracting DCs, macrophages and B-lymphocytes which act as antigen-presenting cells (APC). A greater amount of DCs and macrophages phagocyte FMDV, process it and express the antigens on its membrane with major histocompatibility complex (MHC) intervention, these being recognized by Th-lymphocytes, giving rise to Th1 and Th2 differentiation. The cytokines secreted by these cells stimulate macrophage, neutrophil, DC, natural killer cell, CTL, and B-lymphocyte proliferation and differentiation. The virus’ soluble antigens (SA) bind to B-cell Abs simultaneously with antigen presentation by antigen-presenting cells; this is followed by these cells becoming transformed into plasma cells secreting IgM, IgG, and IgA, where IgM and IgG opsonize viral particles, stimulating phagocytosis, neutralising and activating antibody-dependent cellular cytotoxicity (ADCC), whilst IgA contributes to retaining viral particles in the mucosa, avoiding infection of epithelial cells. Infected target cells also express viral proteins from their cytosol along with MHC class I intervention and CTL are recognized, enabling Th1 stimulus to induce infected cell apoptosis. Antigen binding to B- and T-lymphocyte receptors triggers their activation; this is mediated by a complex cytokine network (interleukins (IL), tumor necrosis factors (TNF), interferons (IFN), colony-stimulating factors (CSF) and chemokines) connecting cells to each other, regulating their function by inducing or supressing their synthesis or that of other cytokines and their receptors. Activated lymphocytes multiply by clonal expansion, producing two types of cell: effector and memory cells. Effector cells have a short lifespan and effect and regulate response to a pathogen. Memory cells live for a long time, increasing immune response speed and intensity if the same pathogen is found again.

A new interferon family, IFN III or IFN-λ, has been described in many species during the last decade, including humans, mice, swine, chicken, and cattle (called boINF-λ3 in the latter) [73]. Although its effectiveness is still being studied, INF-λ-induced antiviral activity has been demonstrated against many viruses, most of them replicating in epithelial cells, such as the influenza virus, respiratory syncytial virus (RSV), human metapneumovirus (hMPV), and coronavirus. Hepatitis B and C, HIV, and herpes simplex viruses have also been shown to be sensitive to IFN-λ activity [75,76] and, as FMDV is a pathogen whose first replication site is the oropharyngeal mucosa, it has been proposed that boIFN-λ3 is expressed in susceptible animals as a mechanism for preventing and protecting them from such viral invasion [73,76] (Figure 3).

Some particularities regarding ruminants’ immune system are not clear, such as the large amount of γδT cells compared to those in humans and mice [48,64] and the situation concerning swine is no less complex [77]. The greater amount of γδT cells has been attributed to a γδT subpopulation expressing the workshop cluster 1 (WC1) molecule in ruminants and its orthologue in swine. This population has only been observed to date in cloven-hooved mammals (Artiodactyla); γδT cells are the first T-lymphocytes to be developed and are found in epithelial cell-associated tissues, such as the skin and intestinal and lung mucosa.

It has been stated that γδT response is directed against pathogens (virus, bacteria, and parasites), reinforcing the immune response at these sites; it has also been observed that they respond to PAMPs, suggesting that they play an important role in the innate immune response and could bridge immune and adaptive responses [78,79]. Research regarding swine has shown that emergency vaccination against FMDV using a high potency vaccine stimulated γδT cells for synthesising cytokines and chemokines, indicating an innate response-related function for these cells in FMDV infection or regarding vaccination against it. These cells were isolated from cattle vaccinated against FMDV and had a cytostatic and cytotoxic effect on the infected cells [80,81].

NK cells are part of the innate immunity mechanism, ensuring the early elimination of pathogen-infected cells and thereby preventing dissemination amongst hosts and healthy individuals. NK cells differentiate infected or damaged cells from healthy ones by activating and inhibitory receptors; these recognize cell surface molecules and promote or inhibit a recognition-type response. Activating receptors detect molecules expressed by infected cells and stimulate lytic activity in the NK which initiates infected cell destruction and the inhibitory receptors identify healthy cells for protecting them [82,83]. The activating receptors located in NK lymphocytes recognize a large variety of ligands; many of these receptors have been called killer cell immunoglobulin (Ig)-like receptors (KIR) since they have a structural domain called Ig-fold (a sandwich-like structure). The adaptive immune system produces IgG1 and IgG3 Abs during infection which bind to the pathogen’s antigens expressed on infected cells and NK receptors bind to the Abs’ Fc regions, activating NK, and these lymphocytes can thus destroy infected cells. This mechanism has been called antibody-dependent cellular cytotoxicity (ADCC) [82,84] (Figure 3).

Much of what is known about NK-mediated immunity against pathogens has come from studies in humans and mice; these cells’ role in domestic animals is still not clear, mainly due to diversity amongst species; however, studies have increased regarding cattle and swine. Bovine NK cells are found in peripheral blood, the liver, lungs, lymph nodes, and bone marrow. As in other species, bovine NK cells express natural cytotoxic receptors such as CD335, produce IFN-γ, lyse sensitive targets and seem to have CD335^+^/CD2^+^^/−^/CD8^+/−^/CD3^−^ phenotypes; NK are identified in swine as CD2^+^/CD8^+^/CD3^−^ [48]. It has been observed that NK-mediated specific responses are greater in infected cattle than in vaccinated ones when trying to understand how NK cells function during FMDV infection. In vitro research involving NK cells from FMDV-infected cattle revealed a high level of cytotoxicity but swine NK cells were minimally cytotoxic, even against FMDV-infected cells [64,84,85].

#### 2.4.2. Acquired Response

##### Cellular Response

Studies regarding T-helper (Th) cell role have revealed that the immune response to FMDV is T-dependent and heterotopic, since the T-lymphocytes extracted from infected animals or those vaccinated with FMDV can proliferate in response to other viral strains [86,87].

An important action has been described for CD4^+^ and CD8^+^ T-lymphocytes regarding their role as eliminators of the virus from infected animals’ tissues; CD4^+^ T-lymphocytes recruit and activate macrophages and neutrophils so that they destroy intracellular and some extracellular pathogens and help B-lymphocytes to produce Abs and CD8^+^ T-lymphocytes to carry out their cytotoxic action (killing infected cells and exposing the agent), eliminate pathogens (usually viruses) located in cell cytosol infecting and reproducing in all cells, including non-phagocytic ones [2,3,5]. Regarding FMDV, T-lymphocyte action has been seen during infection and vaccination in both cattle and swine; however, the response has been greater in infected rather than vaccinated animals. CD4^+^ T-lymphocyte proliferation has been demonstrated and associated with increased neutralising antibody (nAb) production and part of the CD8^+^ T-lymphocyte response. Swine infected by FMDV field strain A24 have induced a CD8^+^ T-lymphocyte-specific response; it is thought that this response is directed towards FMDV A24-specific CD8^+^ epitopes (such epitopes having recently been identified in cattle) [85,88].

Childestone et al. [85] observed anti-FMDV CD8^+^ activity five weeks pi, having proposed that CD8^+^ lymphocytes participate during the infection’s late phase, bearing in mind that FMDV is a cytopathic virus, and it is thus unlikely that a CD8^+^-type response would occur during the disease’s acute phase. A protection-inducing response during this stage is mainly Ab-mediated, neutralising activity playing a predominant role; however, CD8^+^ lymphocytes could play a more important role during the late stage [85] (Figure 3).

CD4^+^ and CD8^+^ T-lymphocyte antigen recognition is associated with the major histocompatibility complex (MHC), called bovine leucocyte antigen (BoLA) for cattle and swine leucocyte antigen (SLA) in pigs. It is an important genetic region influencing resistance to diseases and its level of polymorphism is associated with host immune capability. Class I and II genes are the most polymorphic ones; regarding class II genes, the DRB gene is located in region IIa, along with its loci: DRB1, DRB2 and DRB3. The polymorphism of DRB3 exon 2 (encoding the peptide-binding region) has been related to variability in the immune response to FMDV inactivated vaccine serotypes O, A and ASIA 1. Gowane et al. [89] detected eleven alleles having more than 3% frequency in the population being studied and determined that alleles DRB3/0201/0801 and 1501 always ranked high for the protective immune response [89]. Class I molecules present protein antigen-derived peptides to CD8^+^ T-lymphocytes (which they recognize) and class II molecules present peptides to CD4^+^ T-lymphocytes [90,91,92].

##### Humoral Response

Cell and antibody (Ab)-mediated immunity is an essential part of host control of natural infection; a virus is neutralized within a host by Ab-dependent mechanisms similar to those occurring in neutralization in vitro; however, it has been suggested that macrophages play an essential role in clearing the virus from an animal’s tissues via opsonization-enhanced phagocytosis [48,93].

The rapid induction of high IgM nAb titres (2–4 days pi) is characteristic of responses against cytopathic viruses, like FMDV, usually produced in a thymus-independent form. These Abs are mainly responsible for controlling infection since they prevent the virus’ systemic dissemination [94] (Figure 3). A second strong response by nAbs occurs following field infection with FMDV, taking precedence over recovery; such immunity is against a virus having the same serotype, is prolonged and produced 7 to 14 days pi (IgG). The production of non-protection-inducing Abs against VP2 and VP3 protein antigenic sites has also been reported, acting against the viral capsid’s pentamer subunit and against non-structural proteins, especially RNA polymerase [95]. Following vaccination with inactivated virus, only Ab-mediated humoral immunity is activated and Abs are produced which neutralize infection by the viral serotypes included in the vaccine [87]. It has been observed that protection duration, nAb titres, and vaccine-induced Ab responses are always lower than those induced by natural infection and that simply the presence of nAbs (in many cases) is not enough for ensuring protection. This could indicate that cellular immunity would play an important role in controlling the disease [96].

Mulcay et al. [97] have demonstrated IgG subclass dynamics. High IgG1 levels were seen when evaluating anti-FMDV vaccine protection which, in vitro, were more efficient for complement fixation than IgG2 subclass, and equally for high affinity interaction with phagocyte Fc receptors, thereby promoting opsonization and phagocytosis by antigen-Ab complex, providing protection far beyond their neutralising ability (Figure 3).

Results to date have indicated that humoral responses following systemic vaccination against FMD share similarities with that already reported for oral–nasal infection by FMDV in terms of temporal evolution and isotype profile progression. One study highlighted the finding that the oral–nasal infection of vaccinated steers did not give rise to the disease but was followed by a slight fall in nAb titres, correlated with a transitory reduction of FMDV-specific IgM and IgG1 Abs [98].

MALT’s main function is the production of IgA secreted by sensitized lymphocytes. Another important characteristic concerns IgA being secreted regardless of its serum synthesis; its function is to impede germs adhering to and entering the mucosa and a harmful inflammatory response is avoided by the complement pathway not being activated [59]. When IgA binds to a viral particle it prevents its binding to a host cell by blocking specific receptors and this can happen within an epithelial cell when transporting IgA [60] (Figure 3).

Figure 3 summarizes the expected immune response by cattle and/or swine immunological systems against FMDV post-vaccination or post-recovery from field infection.

### 2.5. Evasion Mechanisms

FMDV is very small, having a 25 to 30 nm diameter, thereby enabling particles to evade the upper respiratory tract’s physical barriers and pass from the upper airways to the lower ones [99]. Even though macrophage, DC and polymorphonuclear infection mechanisms (via Fc receptors) have been proven in RNA viruses (such as flavivirus), it has been suggested that they have been adopted by FMDV and other picornaviruses, facilitating the virus becoming established in the lower airways [37,38].

Lymphopenia has been observed in swine, followed by FMDV infection, depending on serotype and virulence; lymphopenia has not been detected in cattle; however, a constant amount of leucocytes within normal physiological ranges has been observed during the disease’s period of viraemia and clinical phase. Leucocytes have been evaluated in studies like that by Bautista et al. [89] but no changes have been observed in them; by contrast, evaluation in swine has revealed a significant reduction in CD4^+^ and CD8^+^ cells [68,100].

2B and 2C protein expression has been associated with blocking protein secretion in host cells and increased plasmatic membrane permeability [45,46]. The L^pro^ protein is essential for rapid FMDV genome replication since it is involved in autocleavage of nascent peptide and inactivates eukaryotic translation initiation factor 4F (eIF-4G) which is fundamental for host cell mRNA translation. L^pro^’s function is to inhibit host cell protein synthesis and promote viral protein synthesis, thereby compromising host cells’ ability to develop an antiviral response due to their inability to synthesize new protein molecules [42,66,101]. For example, blocking host cell IFN-α and IFN-β expression promotes the virus’ rapid multiplication and propagation [102].

P3C^pro^ or 3C^pro^ viral proteins have been associated with cell protein cleavage, even though it is still not clear whether they act directly or indirectly in activating cell proteases. Immunofluorescence studies have demonstrated that 3C^pro^ is only found in infected cell cytoplasm [19]. Capozzo [103] demonstrated that this protein is concentrated in the perinuclear region and its expression induces H3 histone cleavage thereby reducing host mRNA levels. Briones et al. [104] described 3CD′ (a 3C^pro^ precursor) in FMDV-infected cells’ nuclei and that this could also produce H3 cleavage, contributing even more towards inhibiting host cell RNA synthesis [19]. Wang et al. [62] have shown that this protein can impede IFN type I receptor signalling, specifically, STAT1 and STAT2 nuclear translocation. These molecules have hundreds of possible transcriptional objectives, including many antiviral effectors and immunostimulatory genes [29,51].

**Figure 4:**
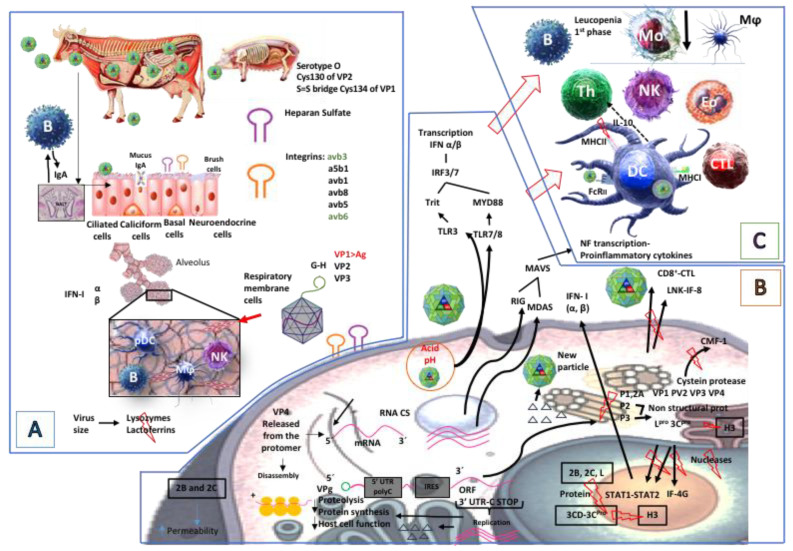
Foot-and-mouth disease virus mechanisms for evading an immune response. (**A**) FMDV enters the airways, taking advantage of its small size which seems to enable it to evade the airways’ lysozymes and lactoferrins. It enters the epithelial cells by receptor-mediated endocytosis: i.e., integrins and heparan sulphate (HS). Capsid protein viral polypeptide 1 (VP1) is the most antigenic viral protein; a region in this polypeptide’s G-H loop consists of 140 to 160 aa; it has been identified as the predominant epitope stimulating nAb production by B-cells. Comparing the structures of various FMDV serotypes has shown that the main differences regarding formation are produced in VP1, VP2, and VP3 loop and C-terminal region, thereby contributing to the virus’ antigenic variation. Dynamic simulations of FMDV’s molecular structure have revealed that the G-H loop protrudes from the capsid’s surface and actively fluctuates as a tentacle in its natural state, suggesting that such “flexibility” could ensure the virus’ correct binding to Abs and cell receptors. The VP2 polypeptide’s Cys 130 region of the virus’ O serotype could covalently bind to VP1 Cys 134 region using a disulphide bond for increasing the extent of variation in the VP1 loop. (**B**) The endosome’s low pH promotes the viral genome’s stripping and release which becomes translocated to host cell cytosol. Positive-sense genomic RNA (gRNA) functions as messenger RNA (mRNA); it has the VPg protein at the 5′ extreme, followed by the 5UTR region with the PolyC region and internal ribosome entry site (IRES) which binds to the ribosomes, followed by the open reading frame (ORF). The 3UTR region and the PolyA tail are at the 3′ extreme. Proteinase L (L^pro^) and P1, P2, and P3 polypeptides are released by viral polyprotein processing by proteolytic cleavage; P1 encodes structural proteins VP1, VP2, VP3, and VP4 which assemble to form the viral capsid. P2 encodes three non-structural proteins: 2A, 2B, and 2C. P3 encodes four non-structural proteins: 3A, 3B, 3C, and 3D. 2B and 2C expression is related to blocking host cell protein secretion and greater permeability of their plasmatic membrane. L^pro^ promotes rapid replication of the viral genome, inactivates (eIF-4G) host cell mRNA translation, inhibits host cell protein synthesis, blocks IFNα and IFNβ expression and promotes viral protein synthesis thereby reducing host cell ability to develop an antiviral response. Viral proteins P3C^pro^ or 3C^pro^ are related to cell protein cleavage in infected cell cytoplasm, induce H3 cleavage, lowering host cell mRNA levels. When 3CD or 3Cpro are found in host cell nucleus this inhibits H3, reducing host cell mRNA synthesis even more so. This could also impede IFN I signalling by altering STAT 1 and STAT 2 nuclear translocation, affecting antiviral effectors and immunostimulatory genes. (**C**). It has been estimated that FMDV also affects the mechanism for antigen presentation by DCs and monocytes; these cells increase IL-10 production, reduce MHC II expression which decreases antigen processing by these cells.

Tumor cell line studies have detected alterations in cell signalling induced by FMDV virion protein 1 (VP1) binding to target cells, thereby causing apoptosis in tumor cells by integrins modulating Akt signalling pathways and reducing COX-2 over-expression and thus tumor progression. Studies have also been evaluating whether FMDV infection reduces the inflammatory response [51,105]. Taken together, these studies have highlighted the varied and powerful way in which FMDV manipulates a host’s immune system to enable its replication and propagation (Figure 4 graphically summarizes such mechanisms).

## 3. Conclusions and Perspectives

Despite reports in the pertinent literature about FMDV’s immunological aspects [6,64,106], this document has contributed towards constructing knowledge in the immunological area, specifically regarding pathogen evasion. It is clear that reducing outbreaks and viral permanency in the environment requires the use of strategies such as mass vaccination of the population in combination with other control measures, thereby enabling Europe and North America to be currently considered FMDV-free [61,107]. In fact, revising prioritization models about the disease in Europe, control measure scores are low, specifically for vaccines (–1), being considered good (www.discontools.eu), and have managed to reduce FMDV incidence. Nevertheless, there are reports of cases in regions having regular vaccination [108] since limited virus replication in the oropharynx is one of the limitations of vaccination enabling vaccinated animals to become infected, making them carriers for a long period of time. The virus’ biological characteristics, mainly regarding its biology (i.e., genetic derivation/drift and recombination), make controlling the disease a greater challenge [107].

Many studies have stressed the significance of FMDV’s infection and evasion mechanisms as one of the greatest challenges for protecting susceptible production systems, starting from vaccination. Studying the innate immune response may cast the greatest light on this mechanism and answer plentiful questions about FMDV. Toka and Golde’s studies in 2013 have clearly shown that early protection can be achieved with very little Ab production, indicating that the humoral immune response plays an important role during the infection’s early phase and should be evaluated in greater depth.

DC and pDC mechanisms of action (MoA), along with IFN I production and variation regarding the response to FMDV amongst bovine and swine species, leads to it being thought that such expression is also key to a better understanding of the innate immune response. FMDV’s ability to infect DC and antigen presentation by them and stimulating the immune response is still poorly understood.

Although advances have been made regarding the virus’ structure and its structural and non-structural proteins’ relationship with target cells, questions still remain unanswered about the pathways and/or mechanisms by which genetic material is released from the endosomes to the cytoplasm.

FMDV has developed mechanisms for breaking or overcoming antiviral response induction and activation, using its non-structural proteins for survival and replicating itself in host cells. L^pro^ and 3C^pro^ are the main proteins modifying host immune responses; L^pro^ has been one of the most studied proteins. It can cleave many host proteins, inhibit cell protein expression and deubiquitinate some crucial molecules for activating antiviral pathways and signal transduction. However, some questions still need to be answered about the different forms of L^pro^ and the pathways involved in L^pro^-mediated antagonist effects. More studies are needed for clarifying such questions and the multifunctional role of L^pro^ in FMDV infection.

FMDV control and prevention programmes can be improved via better understanding and in-depth knowledge concerning pertinent host immune response mechanisms, as well as the virus’ evasion mechanisms; this would include producing new vaccines contributing towards such virus control having wider and better coverage regarding susceptible species.
